# Minimum 15-year results of metasul 28-mm metal-on-metal total hip arthroplasty in patients younger than 50 years of age

**DOI:** 10.1186/s13018-021-02352-2

**Published:** 2021-03-25

**Authors:** Sheng-Yu Jin, Jing-Yao Jin, Joon-Kyoo Kang, Taek-Rim Yoon, Kyung-Soon Park

**Affiliations:** Department of Orthopedics, Center for Joint Disease, Chonnam National University Hwasun Hospital, 322, Seo Yang-Ro, Hwasun-Eup, Hwasun-Gun, Jeonnam 519-809 Republic of Korea

**Keywords:** Metal-on-metal, Long-term result, Cementless total hip arthroplasty, Survivorship

## Abstract

**Background:**

Some propitious mid- and long-term studies had been reported for MoM bearings; however, most studies have addressed specific patient groups rather than younger, active patients, who probably represent the most suitable population for investigations on wear and osteolysis. The purpose of this study to evaluate the long-term results of second-generation metal-on-metal cementless total hip arthroplasty (THA) in patients aged <50 years.

**Methods:**

From December 1997 to January 2004, primary THA using a metal-on-metal bearing cementless implant was performed in 63 patients (72 hips) aged <50 years. The mean follow-up duration was 18.6 (range, 15.9–22.1) years, and the mean age at initial operation was 39 (range, 22–49) years. Clinical results, complications, survivorship, osteolysis, and aseptic loosening were evaluated.

**Results:**

The mean Harris hip score and Western Ontario and McMaster Universities Arthritis Index scores were improved from 57.8 (range, 28–69) points and 73.4 (range, 63–94) points preoperatively to 91.7 (range, 80–100) points and 25.5 points (range, 17–38) points, respectively, at the last follow-up. Osteolysis lesions were found in 12 hips (acetabulum, 6 and femur, 6). The notching occurred on the femoral stem neck occurred in 12 hips. The mean serum cobalt and chromium concentrations were 2.3 (range, 0.2–10.6) μg/L and 1.7 (range, 0.4–8.1) μg/L, respectively, at a mean follow-up of 12.7 years in 32 patients (50.1%). The Kaplan-Meier survivorship curve analysis with revision for any reason as the endpoint revealed that 93.1% survived at 18.6 years’ follow-up.

**Conclusions:**

Second-generation metal-on-metal cementless THA was found to produce satisfactory clinical and radiographic results with a low revision rate for osteolysis and aseptic loosening in patients aged less than 50 years.

## Background

The number of primary total hip arthroplasty (THA) cases is increasing because of the general increase in life span and the expansion of the surgical indications for THA. However, despite the continued improvements in materials, the behaviors of metal-on-metal (MoM) articulation in young, active patients remain uncertain. A study by the Swedish National Hip Registry showed that aseptic loosening was the primary cause of failure of revision after THA in patients aged <50 years [1]. Alternative bearing components have been proposed for patients at greater risks of wear and osteolysis, and ceramic-on-ceramic articulations are known to have better wear resistance than MoM articulations [2]. However, ceramics had the disadvantage of breakage, and ceramic bearings were associated with earlier revision than other bearings and sensitive to problems, such as impingement and squeaking [3]. In the late 1980s, the introduction of the second-generation metallic bearing components generated renewed interest in MoM THA [4] owing to adequate and reproducible clearance.

Some propitious mid- and long-term results have been reported for MoM units [5–16]; however, most studies have addressed specific patient groups rather than younger, active patients, who probably represent the most suitable population for investigations on wear and osteolysis.

In the present study, we evaluated the long-term clinical and radiographic results and implant survivorship of using second-generation MoM cementless THA in patients aged <50 years.

## Materials and methods

### Patients

**Fig. 1:**
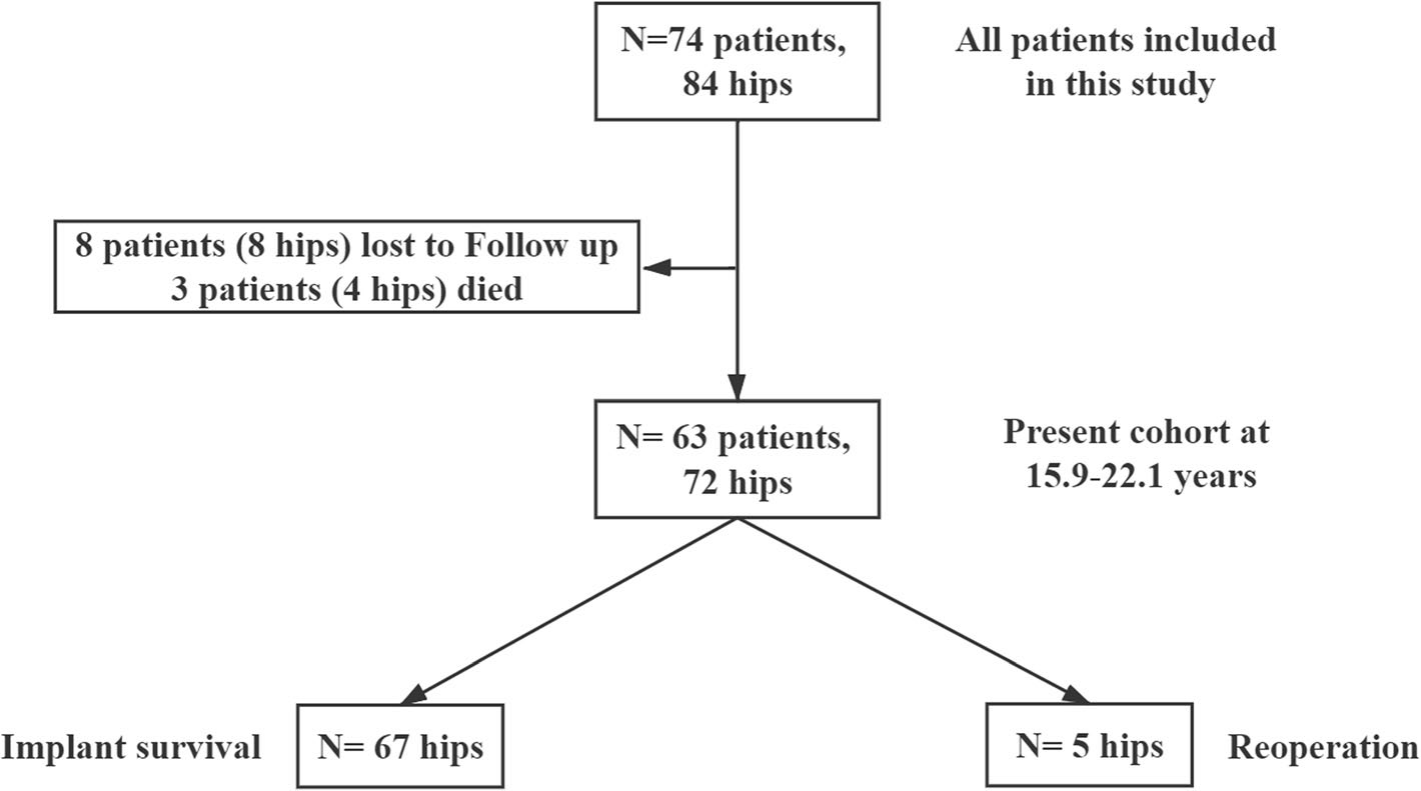
Patient flow diagram

The study cohort included 74 patients (84 hips) aged <50 years who underwent primary cementless THA with second-generation MoM bearings at our institute between December 1997 and January 2004. Eight patients (8 hips) were lost to follow-up, and 3 patients (4 hips) died for reasons unrelated to the primary surgery before reaching 15 years of age. The remaining 63 patients (72 hips) were evaluated for clinical outcomes (Fig. 1). The study group included 43 men (50 hips) and 20 women (22 hips), with an overall mean age of 39 (range, 22–49) years at operation. The mean follow-up duration was 18.6 (range, 15.9–22.1) years (Table 1). The decision to use a MoM bearing depended on the patient’s preference after being informed of the specific benefits and risks of bearing surfaces.

**Table 1:** Demographics of the patients

Parameters	
Number of patients (hips)	63 (72)
Gender (male/female)	43/20
Age (years)	39 (22–49)
Mean follow-up duration	18.6 (15.9–22.1)
Body mass index (kg/m^2^)	23.4 (16.6–38.3)
Diagnosis	
Osteonecrosis	37
Osteoarthritis	13
Septic hip sequelae	7
Rheumatoid arthritis	5
Femoral neck fracture	3
Tuberculous coxitis	3
Pyogenic coxitis	2
Femoral head fracture dislocation	1
Ankylosed hip	1
Preoperative HHS	58.9 (35-69)
Preoperative WOMAC score	72.2 (63-94)

*WOMAC* Western Ontario and McMaster Universities Osteoarthritis Index, *HHS* Harris hip score

The diagnoses were osteonecrosis of the femoral head in 37 hips, osteoarthritis in 13 hips, septic hip sequelae in 7 hips, rheumatoid arthritis in 5 hips, femoral neck fracture in 3 hips, tuberculous coxitis in 3 hips, pyogenic coxitis in 2 hips, femoral head fracture-dislocation in 1 hip, and ankylosis in 1 hip (Table 1).

All the procedures were performed by a single surgeon using a posterolateral approach in the lateral position. In all the cases, a Fitmore acetabular cup (Zimmer, Warsaw, IN, USA), 28-mm Metasul metal bearing (Zimmer, Warsaw, IN, USA) were used. The Metasul metal bearing system contained that high-carbon femoral head and metal inlay polyethylene liner, which forged in high carbide cobalt-chromium alloy. Two types of femoral stems were used, namely the CLS stem (Zimmer, Warsaw, IN, USA) in 48 hips and the Wagner Cone Stem prosthesis (Zimmer, Warsaw, IN, USA) in 24 hips. Postoperatively, an abduction pillow was used between the legs for 4 weeks when in bed to reduce the risk of dislocation, and elastic stockings were used to minimize the risk of deep vein thrombosis. Mobilization was recommended with weight-bearing, as tolerated, on postoperative day 1. The patients were discharged when they were already able to use walking assists properly.

### Clinical and radiographic analyses

The patients were followed up at 6 weeks and 3, 6, and 12 months and then annually thereafter. Two of the authors who were not involved in the surgery checked the Harris hip score (HHS) [17] and Western Ontario and McMaster Universities Arthritis Index (WOMAC) [18] scores at the follow-up visits. An anteroposterior pelvic radiograph that included the proximal part of the femur and the entire stem was taken at each clinical evaluation. The 2 abovementioned independent observers examined all the radiographs. Osteolysis was assessed using the zones described by Gruen et al. [19] or the method described by DeLee and Charnley [20] on anteroposterior pelvic radiographs. To be counted, radiolucent lines adjacent to the prosthesis must occupy at least 50% of the respective zone. Osteolysis was defined as described by Zicat et al. [21] as a focal radiolucent area ≥ 2-mm wide at the final follow-up that was not evident on the immediate postoperative radiographs. Complications were evaluated at each follow-up visit.

Moreover, metal ion concentration tests were performed for the patients. The serum cobalt concentrations were measured with atomic absorption spectrophotometry (Varian, Victoria, Australia) with a detection limit of 0.1 μg/L, and atomic absorption spectrophotometry (PerkinElmer, Inc, Boston, MA) with a detection limit of 0.1 μg/L was used to measure the serum chromium concentrations.

The paired Student’s *t* test was used to analyze the differences between the outcomes. The chi-square test was used to compare categorical values. The Pearson correlation coefficient was used to identify any correlation between the multiple continuous variables (e.g., age, body mass index [BMI], cup inclination, and anteversion) and notching. Binary logistic regression was used to determine the correlation between the categorical variables (e.g., sex, diagnosis, and types of femoral stem) and notching. Implant survival was assessed using the Kaplan-Meier analysis with revision for any reason or aseptic failures as the endpoint. All statistical analyses were performed using the SPSS version 25.0 statistical software system (SPSS Inc., Chicago, Ill), and *p* values <0.05 were considered significant.

## Results

### Clinical and radiographic outcomes

After >15 years of follow-up, the mean Harris hip score improved from 57.8 (range, 28–69) points to 91.7 (range, 80–100) points, and the mean WOMAC score improved from 73.4 (range, 63–94) points to 25.5 (range, 17–38) points. The mean cup inclination and anteversion angles were 39.0 (range, 29–55)° and 21.7 (range, 10–28)°, respectively. The mean duration of the operation was 77 min (range, 65–95 min). The mean total blood loss was 518.2 mL (range, 280–790mL).

### Osteolysis around the THA

Osteolysis around the cup was observed in 6 (8.3%) of the 72 hips. A radiolucent line and osteolysis around the stem were observed in 6 (8.3%) of the 72 hips and were located in Gruen zone 1 in 4 hips, Gruen zone 7 in 1 hip, and Gruen zones 1 and 7 in 1 hip. No cup or stem was considered loose owing to a radiolucent line of >2-mm thick or to occupy ≥2 DeLee and Charnley zones. All details of osteolytic lesions showed in Table 2.

**Table 2:** Osteolytic lesions around the THA

DeLee and Chanley zones - acetabular osteolysis	Number of hips
Zone 2	2 hips
Zone 3	3 hips
Zones 1 and 2	1 hip
Gruen zones - femoral osteolysis	Number of hips
Zone 1	4 hips
Zone 7	1 hip
Zones 1 and 7	1 hip

*THA* Total hip arthroplasty

**Fig. 2:**
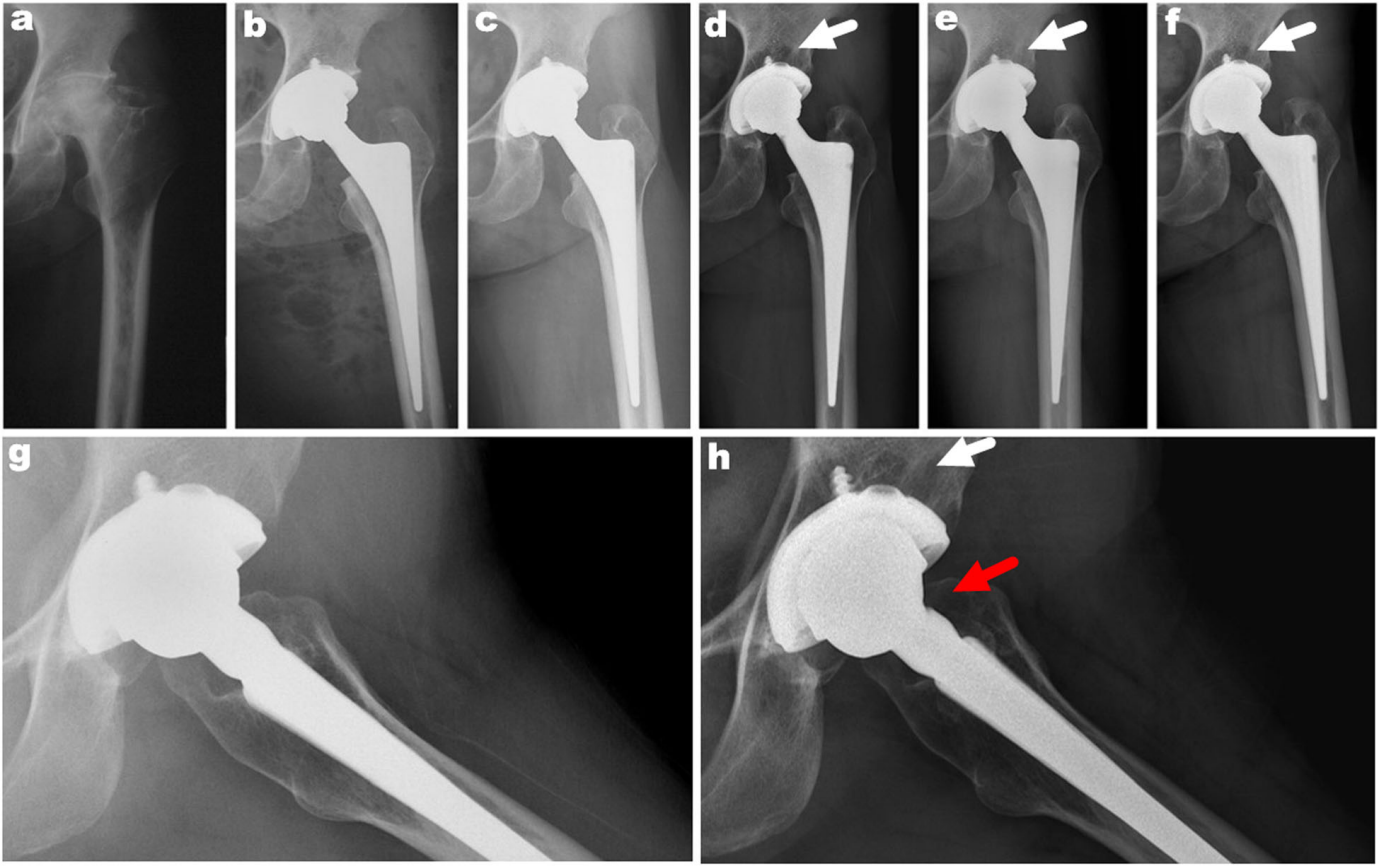
**a** Preoperative anteroposterior radiograph of the left hip showing avascular necrosis of the femoral head. **b** Immediate postoperative anteroposterior radiograph showing a well-fixated metal-on-metal articulated total hip arthroplasty. **c**–**f** An anteroposterior radiograph of the right hip at 3, 5, 11, and 22 years after operation, 5 years after surgery we can see osteolysis around the cup (white arrow). **g** Immediate postoperative frog-view radiograph. **h** A frog-view radiograph of the right hip at 22 years after operation, showing an obvious notch at the anterior part of the neck of the femoral stem (red arrow)

Notching in the neck of the femoral stem was observed in 12 cases (12/72, 16.7%). Eleven notchings occurred at the anterior area of the femoral stem (Fig. 2), and 1 was in the medial-superior part of the neck of the femoral stem. In the correlation analysis, no significant correlations were found between notching on the femoral stem and age (*p* = 0.085), sex (*p* = 0.819), diagnosis (*p* = 0.483), BMI (*p* = 0.552), types of femoral stem (*p* = 0.998), cup inclination (*p* = 0.107), and anteversion (*p* = 0.475) (Table 3).

**Table 3:** Correlation analysis of factors for notching of the femoral stem neck

Variables	*p* values
Age	0.085*
Sex	0.819†
Diagnosis	0.483†
Body mass index	0.552†
Types of femoral stem	0.998†
Cup inclination	0.107*
Cup anteversion	0.475*

*Results of Pearson correlation coefficient. ^†^Results of univariate binary logistic regression

Fifteen patients were excluded from serum ion concentration measurements owing to complications, such as infection, dislocation, periprosthetic femoral fractures, cobalt- or chromium-containing fixation devices, and chronic renal failure or insufficiency; Sixteen patients refused to have their serum ion concentrations assessed. The remaining 32 patients (50.1%) underwent routine examinations to assess their serum cobalt and chromium concentrations. At the mean 12.7-year postoperative follow-up, the mean serum cobalt and chromium concentrations were 2.3 (range, 0.2–10. 6) μg/L and 1.7 (range, 0.4–8.1) μg/L, respectively. The mean serum cobalt and chromium concentrations were 2.2 (range, 0.2–10.6) μg/L and 1.6 (range, 0.4–8.1) μg/L, respectively, in the non-notching group and 2.5 (range, 0.8–5.1) μg/L (*p* = 0.652) and 2.3 (range, 0.6–5.5) μg/L (*p* = 0.343), respectively, in the notching group.

### Implant survivorship

**Fig. 3:**
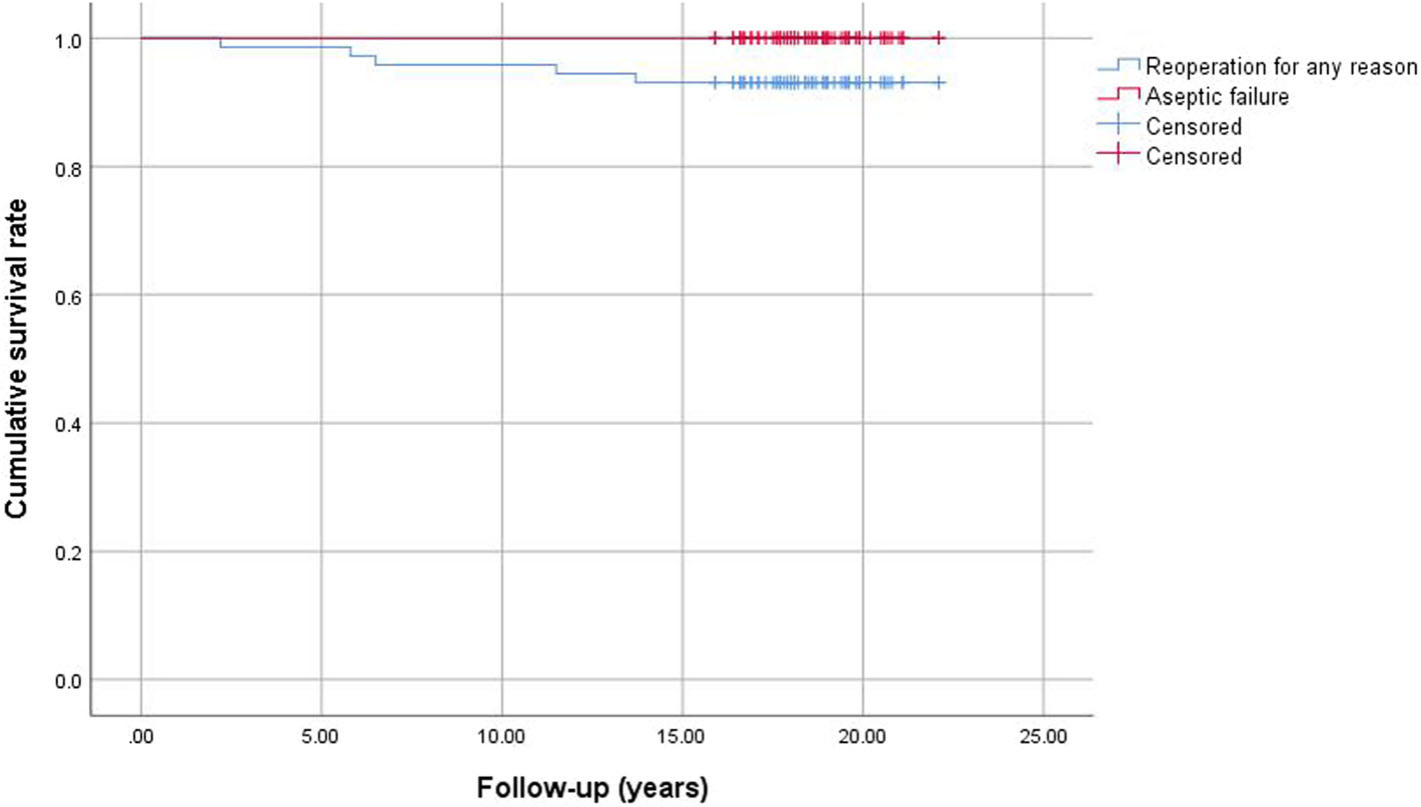
**a** Kaplan-Meier survival curve for reoperation for any reason as the endpoint. **b** Kaplan-Meier survival curve for aseptic failure

The Kaplan-Meier survivorship curve analysis with reoperation for any reason as the endpoint revealed a survival rate of 93.1% (95% confidence interval, 84.5–97.7%) at 18.6 years (Fig. 3). The Kaplan-Meier survivorship analysis with revision for aseptic failure as the endpoint revealed a survival rate of 100% (Fig. 3).

### Revisions

**Fig. 4:**
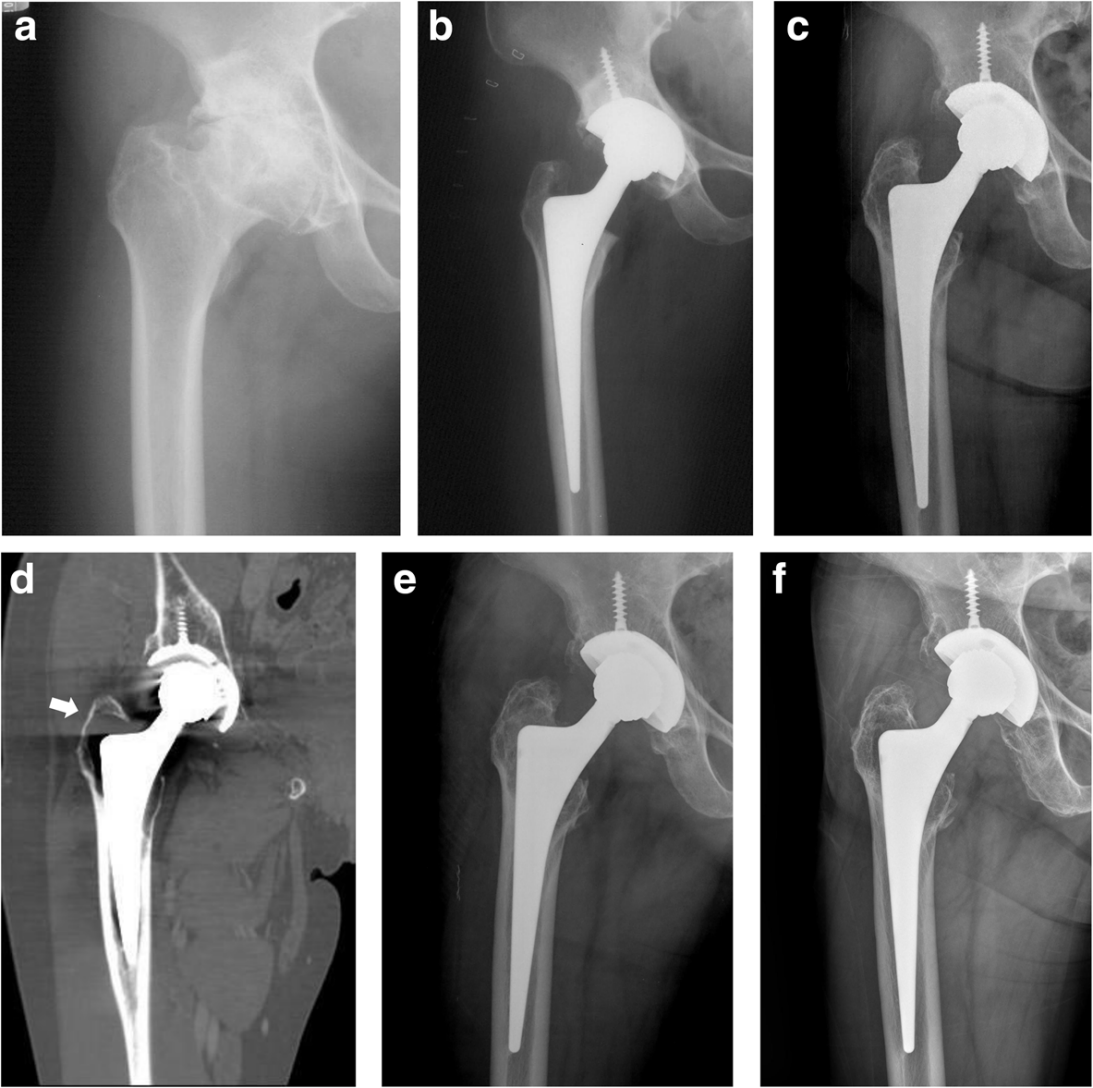
Osteolysis around the femoral stem. **a** Preoperative anteroposterior radiograph of the right hip showing osteoarthritis. **b** Immediate postoperative anteroposterior radiograph showing a well-fixated metal-on-metal articulated total hip arthroplasty. **c**–**d** An anteroposterior radiograph of the right hip at 11 years after operation. Computed tomography (coronal view) image before revision, showing an osteolysis around the proximal femoral stem, especially the greater trochanter (white arrow). **e** One year after revision with bone grafting around the greater trochanter. **f** An anteroposterior radiograph of the right hip at 21 years after total hip arthroplasty

The postoperative complications were acute postoperative infection in 1 hip (1.4%), delayed infection in 1 hip (1.4%), dislocation in 3 hips (4.2%), periprosthetic femoral fractures in 2 hips (2.8%), and greater trochanteric osteolysis destruction in 1 hip (1.4%). Delayed infection occurred at 5.6 years after THA and was treated with curettage, debridement, and liner change without acetabular or femoral component revision. Of the 3 cases with hip dislocation, 2 with early hip dislocation were treated with closed reduction, and 1 with late hip dislocation after 6.5 years postoperatively required revision with a liner change, use of a larger head, and posterior soft tissue augmentation with reapproximation of the posterior capsule and the short external rotators to the posterior aspect of the greater trochanter. No implant instability was observed at the final follow-up. Of 2 cases of periprosthetic femoral fracture, one was classified as Vancouver B1 after 2 years postoperatively, which was treated with internal fixation with a limited-contact dynamic compression plate (LC-DCP) and cables, and the other was sustained a Vancouver B2 fracture at 13 years after THA, which required a stem revision along with plate fixation using a Wagner SL revision hip stem (Zimmer, Warsaw, IN, USA). One case with lateral hip pain and greater trochanteric osteolysis destruction after 11 years postoperatively was reoperated with isolated bone grafting (Fig. 4).

## Discussion

The purpose of the present study was to assess the long-term outcomes of second-generation MoM cementless THA in patients aged <50 years. The main concern of THA in younger patients is implant longevity. The Annual Report of the Swedish Hip Arthroplasty Registry (2013) concluded that the age factor seriously affects the survival rate in various types of primary THA [22]. In our study, the survival rate in 28-mm MoM cementless THA for any revision was 93.1% at 18.6 years, indicating that the MoM bearing is still a suitable choice for patients aged <50 years.

A previous study reported that the risk of aseptic loosening decreased by 1.8% per year of patient age [23]. Polyethylene wear and subsequent osteolysis of metal-on-polyethylene bearing THA prostheses were the main factors that compromised the long-term total joint replacement survival of younger patients [24]. This osteolysis is the result of biological reactions caused by wear particles from polyethylene liners within the acetabulum [25] because the amount of wear debris in the joint articulation induced inflammatory responses [26], which can result in significant osteolysis even when a liner is not excessively worn. Furthermore, although metal-on-conventional polyethylene bearing THA is the appropriate choice for older, less active patients [27], THA has been shown to consistently provide inferior results and high rates of loosening in younger patients [28]. Kim et al. reported that 149 cups (16%) were revised for polyethylene bearing wear and osteolysis loosening in more active young patients (mean age, 39.3 years) with a minimum follow-up of 10 years. They found that the high rates of osteolysis and wear were major challenges [29]. Adelani et al. [30] reviewed a cohort of 71 patients aged ≤55 years who underwent THA for revision. The use of the conventional polyethylene bearing was associated with major failure after revision. They advised against using the conventional polyethylene bearing in revision surgeries because the wear particles from polyethylene accumulate locally over time and cause severe osteolysis. In our study, the rate of metal particle-induced osteolysis around either the cup or the stem was 15.3% on the final radiographs. However, 1 case alone had a revision with bone graft surgery due to osteolysis at the area of the greater trochanter.

Multiple mid- and long-term follow-up studies have reported favorable results and good survivorship with 28-mm head MoM articulations. Innmann et al. [31] reported a cohort of 91 patients (100 hips) aged <50 years who underwent cementless THA using Metasul MoM articulations. During the 18.6-year follow-up, none of the cases had loosening or osteolysis around the cup. One hip alone had a revision due to late aseptic loosening of an undersized stem. Aseptic loosening was not a primary cause of revision in their study. Moon et al. [13] investigated outcomes at a mean follow-up of 20 years in 92 patients treated with a Metasul MoM articulation and an uncemented modular acetabular component. Twelve cases (10.5%) were identified as osteolysis around the cup and 7 (6.1%) as osteolysis around the stem. Ten hips (8.8%) required revision surgery for aseptic loosening with osteolysis. This MoM bearing showed an excellent survival rate during the 20-year mean follow-up. These results showed that the clinical outcomes of 28-mm MoM articulations are excellent in terms of durability and that the incidence of osteolysis in these hips was much lower than that in hips with a polyethylene bearing surface. The rate of aseptic loosening failure in our study was comparable with the abovementioned findings. The incidence of osteolysis around the cup and the stem at final follow–up was 8.3% and 8.3%, respectively. However, none of these cases with osteolytic lesions made a failure of the implant with loosening even during the mean follow-up of 18.6 years.

The results in the present study are comparable to the published findings by Biemond et al. [32], who reported that four hips (4%) required revision due to femoral stem aseptic loosening during a mean follow-up of 18.4 years. Faldini et al. [33] retrospectively reviewed a group of 28 THAs with the Wagner Cone Prosthesis in patients younger than 50 years old. After a 12-year follow-up, no evidence of loosening was observed, and none of the femoral stems has been revised. Consistent with previous studies [32, 33], high-intensity physical activity in patients aged <50 years did not affect the implant survivorship.

Nevertheless, despite these clinical advantages, the use of MoM bearing remains an issue of concern, and adverse local soft tissue reactions specific to metal particulate debris caused by MoM articulations have been reported [34]. However, most of these reports were based on larger-diameter THA and MoM hip resurfacing arthroplasty [35–37]. In a study on the frequency of adverse reactions to metal debris (ARMD) after MoM THA from 2000 to 2011, 1.2% (160/12,961) of the cases were reported to have ARMD [38]. The incidence of ARMD in cases with small head Metasul bearings was 0.5% (7/1535), which is lower than those in other types of devices [38]. Although we found no ARMD in the specific population after long-term follow-up in the present study, most patients showed that either the cobalt or chromium concentration was within the normal range.

In the present study, 12 hips had notching on the neck of the femoral stem. However, none of these cases complained of pain around the hip joint. Among these hips, 5 (41.7%) had osteolytic lesions around the cup. We believe that cross-legged sitting or squatting position, which is more common in Korean populations, is probably responsible for the notching on the anterior part of the neck of the femoral stem due to impingement between the inner metal liner and the neck of the femoral stem. We want to determine whether differences exist in the serum cobalt and chromium concentrations after 28-mm head MoM THA between patients with notching and those without notching. To investigate this, we compared the serum iron concentrations of the notching group with those of the non-notching group. We found no significant differences between the two groups. Meanwhile, the incidence rate of osteolysis was higher in the notching group than in the non-notching group. This can be explained by the material of the femoral neck being titanium, which is softer than the material used for the head bearing surface (a high-carbon, wrought-forged cobalt-chromium alloy). When impingement frequently occurs between the neck and the head surface bearing, the impingement would always occur on the softer neck. Although no evidence showed ARMD in these patients, potential asystematic adverse reactions should be closely monitored during the follow-up of selected patients.

Dislocation is one of the most common complications of THA. Cases with recurrent dislocation frequently require revisions. Late dislocation occurs ≥5 years after THA [39] and is associated with the intrinsic instability of the prosthesis, polyethylene wear, and spinopelvic imbalance [40, 41]. In addition, cases with a greater postoperative range of motion caused by a pseudocapsule could lead to late dislocation. Moreover, a decline in the static soft tissue strength over time can unmask a problem of a malpositioned implant later when it presents as a late dislocation [42]. Theoretically, metal debris generated from the articular surface could cause significant damage to the soft tissue and muscle strengths around the hip over time [43] [44]. Furthermore, neck liner impingement at the maximum range of motion is another factor that causes a high rate of dislocation. In this study, the rate of recurrent dislocation was relatively low at 2.7%, with 1 hip alone requiring revision with a liner change. During the revision, we found that impingement was obvious during the movement, which showed a severed notching caused by the impingement. For young patients with extensive ranges of movement, care should be taken to avoid malpositioning of the implant during operation.

Some limitations of this study should be considered. First, medical records were reviewed retrospectively, a relatively small number of patients with limited follow-up duration were included, and no control group was included. However, this study included a targeted specific population (patients aged <50 years). In addition, we used 2 types of cementless femoral stems according to the proximal femoral geometry in this study. However, our study was focused on the MoM surface bearing, and none of the cases required revision for femoral stem failure. Third, the titanium concentrations were not evaluated during the follow-ups. Notching of the femoral stem neck may be increase the serum titanium concentrations because the femoral stem neck was made of titanium alloy. Furthermore, we suggest that more cases with longer follow-up durations are needed to evaluate metal-specific problems, such as adverse local tissue reactions and delayed hypersensitivity, which could negatively impact long-term outcomes.

## Conclusions

Our study demonstrates that using second-generation MoM cementless THA produces acceptable clinical and radiographic results at a mean follow-up of 18.6 years in patients aged <50 years. The rates of complications associated with the bearing surface and revision for osteolysis and aseptic loosening were low.

## Data Availability

The data sets supporting the results of this article are included within the article and its additional files. The datasets are available from the corresponding author on reasonable request.

## Abbreviations

THA: Total hip arthroplasty
MoM: Metal-on-metal
BMI: Body mass index
HHS: Harris hip score
WOMAC: Western Ontario and McMaster Universities Arthritis Index
LC-DCP: Limited-contact dynamic compression plate

## References

[1] Malchau H, Herberts P, Eisler T, Garellick G, Söderman P (2002). The Swedish total hip replacement register. J Bone Joint Surg Am.

[2] Rieker CB (2016). Tribology of total hip arthroplasty prostheses: what an orthopaedic surgeon should know. EFORT Open Rev.

[3] Migaud H, Putman S, Kern G, Isida R, Girard J, Ramdane N, Delaunay CP, Hamadouche M (2016). Do the reasons for ceramic-on-ceramic revisions differ from other bearings in total hip arthroplasty?. Clin Orthop Relat Res.

[4] Weber BG. Experience with the Metasul total hip bearing system. Clin Orthop Relat Res. 1996;(329 Suppl):S69–77. 10.1097/00003086-199608001-00007. 10.1097/00003086-199608001-00007 8769324

[5] Park CW, Kim JH, Lim SJ, Moon YW, Park YS (2019). A minimum of 15-year results of cementless total hip arthroplasty using a 28-mm metal-on-metal articulation. J Arthroplasty.

[6] Halma JJ, Godefrooij DA, Eshuis R, van Gaalen SM, de Gast A (2014). Excellent survivorship of the Morscher monoblock cup with a 28-mm Metasul-on-Metasul bearing at a mean of 5-year follow-up. J Arthroplasty.

[7] de Steiger R, Peng A, Lewis P, Graves S (2018). What is the long-term survival for primary tha with small-head metal-on-metal bearings?. Clin Orthop Relat Res.

[8] Lass R, Grübl A, Kolb A, Domayer S, Csuk C, Kubista B, Giurea A, Windhager R (2014). Primary cementless total hip arthroplasty with second-generation metal-on-metal bearings: a concise follow-up, at a minimum of seventeen years, of a previous report. J Bone Joint Surg Am.

[9] Hwang KT, Kim YH, Kim YS, Ryu JA (2014). Prevalence of a soft-tissue lesion after small head metal-on-metal total hip replacement: 13- to 19-year follow-up study. Bone Joint J.

[10] Ayoub B, Putman S, Cholewinski P, Paris A, Migaud H, Girard J (2017). Incidence of adverse reactions to metal debris from 28-mm metal-on-metal total hip arthroplasties with minimum 10 years of follow-up: clinical, laboratory, and ultrasound assessment of 44 cases. J Arthroplasty.

[11] Zuiderbaan HA, Visser D, Sierevelt IN, Penders J, Verhart J, Vergroesen DA (2018). Long-term clinical results of the Metasul metal-on-metal total hip arthroplasty: 12.6 years follow-up of 128 primary total hip replacements. Hip Int.

[12] Saito S, Ishii T, Mori S, Hosaka K, Ootaki M, Tokuhashi Y. Long-term results of metasul metal-on-metal total hip arthroplasty. Orthopedics. 2010;33(8). 10.3928/01477447-20100625-11. 10.3928/01477447-20100625-11 20704108

[13] Moon J-K, Kim Y, Hwang K-T, Yang J-H, Oh Y-H, Kim Y-H (2018). Long-term outcomes after metal-on-metal total hip arthroplasty with a 28-mm head: a 17- to 23-year follow-up study of a previous report. The Journal of Arthroplasty.

[14] Moon JK, Kim Y, Hwang KT, Yang JH, Ryu JA, Kim YH (2019). Prevalence and natural course of pseudotumours after small-head metal-on-metal total hip arthroplasty: a minimum 18-year follow-up study of a previous report. Bone Joint J.

[15] Erivan R, Villatte G, Lecointe T, Mulliez A, Descamps S, Boisgard S (2019). Long-term survival of hybrid total hip arthroplasty with the uncemented CLS cup, cemented Müller cobalt-chromium stem, and 28-mm Metasul™ bearings: Retrospective review of 115 hips after a minimum of 17.8 years. Orthop Traumatol Surg Res.

[16] Kim CH, Ryu JJ, Jeong MY, Kim JW, Chang JS, Yoon PW (2019). Serum metal ion levels in cementless metal-on-metal total hip arthroplasty: long-term follow-up trends. J Arthroplasty.

[17] Harris WH (1969). Traumatic arthritis of the hip after dislocation and acetabular fractures: treatment by mold arthroplasty. An end-result study using a new method of result evaluation. J Bone Joint Surg Am.

[18] McConnell S, Kolopack P, Davis AM (2001). The Western Ontario and McMaster Universities Osteoarthritis Index (WOMAC): a review of its utility and measurement properties. Arthritis Rheum.

[19] Gruen TA, McNeice GM, Amstutz HC (1979). “Modes of failure” of cemented stem-type femoral components: a radiographic analysis of loosening. Clin Orthop Relat Res.

[20] DeLee JG, Charnley J (1976). Radiological demarcation of cemented sockets in total hip replacement. Clin Orthop Relat Res.

[21] Zicat B, Engh CA, Gokcen E (1995). Patterns of osteolysis around total hip components inserted with and without cement. J Bone Joint Surg Am.

[22] Garellick G, Kärrholm J, Lindahl H, Malchau H, Rogmark C, Rolfson O (2015). Swedish hip arthroplasty register, annual report 2013. The Swedish Hip Arthroplasty Register.

[23] Münger P, Röder C, Ackermann-Liebrich U, Busato A (2006). Patient-related risk factors leading to aseptic stem loosening in total hip arthroplasty: a case-control study of 5,035 patients. Acta Orthop.

[24] Stambough JB, Rames RD, Pashos GE, Maloney WJ, Martell JM, Clohisy JC (2018). Conventional polyethylene in total hip arthroplasty in young patients: survivorship, wear analysis, and clinical outcomes between 15 and 20 years. J Arthroplasty.

[25] Tsukamoto M, Ohnishi H, Mori T, Kawasaki M, Uchida S, Sakai A (2017). Fifteen-year comparison of wear and osteolysis analysis for cross-linked or conventional polyethylene in cementless total hip arthroplasty for hip dysplasia-a retrospective cohort study. J Arthroplasty.

[26] Khanna R, Ong JL, Oral E, Narayan RJ. Progress in wear resistant materials for total hip arthroplasty. Coatings. 2017;7(7). 10.3390/coatings7070099.

[27] Milošev I, Kovač S, Trebše R, Levašič V, Pišot V (2012). Comparison of ten-year survivorship of hip prostheses with use of conventional polyethylene, metal-on-metal, or ceramic-on-ceramic bearings. J Bone Joint Surg Am.

[28] Atrey A, Wolfstadt JI, Hussain N, Khoshbin A, Ward S, Shahid M, Schemitsch EH, Waddell JP (2018). The ideal total hip replacement bearing surface in the young patient: a prospective randomized trial comparing alumina ceramic-on-ceramic with ceramic-on-conventional polyethylene: 15-year follow-up. The Journal of arthroplasty.

[29] Kim YH, Kim JS, Park JW, Joo JH (2012). Periacetabular osteolysis is the problem in contemporary total hip arthroplasty in young patients. J Arthroplasty.

[30] Adelani MA, Crook K, Barrack RL, Maloney WJ, Clohisy JC (2014). What is the prognosis of revision total hip arthroplasty in patients 55 years and younger?. Clin Orthop Relat Res.

[31] Innmann MM, Gotterbarm T, Kretzer JP, Merle C, Ewerbeck V, Weiss S, Aldinger PR, Streit MR (2014). Minimum ten-year results of a 28-mm metal-on-metal bearing in cementless total hip arthroplasty in patients fifty years of age and younger. Int Orthop.

[32] Biemond JE, Venkatesan S, van Hellemondt GG (2015). Survivorship of the cementless Spotorno femoral component in patients under 50 years of age at a mean follow-up of 18.4 years. Bone Joint J.

[33] Faldini C, Miscione MT, Chehrassan M, Acri F, Pungetti C, d'Amato M, Luciani D, Giannini S (2011). Congenital hip dysplasia treated by total hip arthroplasty using cementless tapered stem in patients younger than 50 years old: results after 12-years follow-up. J Orthop Traumatol.

[34] Kleeman LT, Goltz D, Seyler TM, Mammarappallil JG, Attarian DE, Wellman SS, Bolognesi MP (2018). Association between pseudotumor formation and patient factors in metal-on-metal total hip arthroplasty population. J Arthroplasty.

[35] Lainiala O, Eskelinen A, Elo P, Puolakka T, Korhonen J, Moilanen T (2014). Adverse reaction to metal debris is more common in patients following MoM total hip replacement with a 36 mm femoral head than previously thought: results from a modern MoM follow-up programme. Bone Joint J.

[36] Lombardi AV, Berend KR, Morris MJ, Adams JB, Sneller MA (2015). Large-diameter metal-on-metal total hip arthroplasty: dislocation infrequent but survivorship poor. Clin Orthop Relat Res.

[37] Kiran M, Armstrong C, Shivarathre D, Peter VK (2017). Blood metal ion levels have limited utility in the surveillance of asymptomatic large-head metal-on-metal total hip arthroplasties. J Arthroplasty.

[38] Sugano N, Iida H, Akiyama H, Takatori Y, Nagoya S, Hasegawa M, Kabata T, Hachiya Y, Yasunaga Y (2014). Nationwide investigation into adverse tissue reactions to metal debris after metal-on-metal total hip arthroplasty in Japan. J Orthop Sci.

[39] Aqil A, Shah N (2020). Diagnosis of the failed total hip replacement. J Clin Orthop Trauma.

[40] Heckmann N, McKnight B, Stefl M, Trasolini NA, Ike H, Dorr LD (2018). Late dislocation following total hip arthroplasty: spinopelvic imbalance as a causative factor. J Bone Joint Surg Am.

[41] Hoggett L, Cross C, Helm T (2017). Experience of the posterior lip augmentation device in a regional hip arthroplasty unit as a treatment for recurrent dislocation. J Orthop.

[42] Hannon CP, Cotter EJ, Cooper HJ, Deirmengian CA, Rodriguez JA, Urban RM, Paprosky WG, Jacobs JJ (2020). Adverse local tissue reaction due to mechanically assisted crevice corrosion presenting as late instability following metal-on-polyethylene total hip arthroplasty. J Arthroplasty.

[43] Killampalli VV, Reading AD (2009). Late instability of bilateral metal on metal hip resurfacings due to progressive local tissue effects. Hip Int.

[44] Shah SM, Walter WL, Tai SM, Lorimer MF, de Steiger RN (2017). Late dislocations after total hip arthroplasty: is the bearing a factor?. J Arthroplasty.

